# Outcomes following cardiopulmonary resuscitation in an emergency department of a low- and middle-income country

**DOI:** 10.1186/s12245-018-0200-0

**Published:** 2018-10-01

**Authors:** Umme Salama Moosajee, Syed Ghazanfar Saleem, Sundus Iftikhar, Lubna Samad

**Affiliations:** 1 Center for Essential Surgical and Acute Care, Global Health Directorate, Indus Health Network, 5th Floor, Woodcraft Building, Sector 47, Korangi Creek Road, Karachi, 75300 Pakistan; 2 Emergency Department, The Indus Hospital, Karachi, Pakistan; 3 Indus Hospital Research Center, The Indus Hospital, Karachi, Pakistan

**Keywords:** Cardiopulmonary resuscitation, Return of spontaneous circulation, Survival to discharge

## Abstract

**Background:**

Cardiopulmonary resuscitation (CPR) is a key component of emergency care following cardiac arrest. A better understanding of factors that influence CPR outcomes and their prognostic implications would help guide care. A retrospective analysis of 800 adult patients that sustained an in- or out-of-hospital cardiac arrest and underwent CPR in the emergency department of a tertiary care facility in Karachi, Pakistan, between 2008 and 15 was conducted.

**Methods:**

Patient demographics, clinical history, and CPR characteristics data were collected. Logistic regression model was applied to assess predictors of return of spontaneous circulation and survival to discharge. Analysis was conducted using SPSS v.21.0.

**Results:**

Four hundred sixty-eight patients met the study’s inclusion criteria, and overall return of spontaneous circulation and survival to discharge were achieved in 128 (27.4%) and 35 (7.5%) patients respectively. Mean age of patients sustaining return of spontaneous circulation was 52 years and that of survival to discharge was 49 years. The independent predictors of return of spontaneous circulation included age ≤ 49 years, witnessed arrest, ≤ 30 min interval between collapse-to-start, and 1–4 shocks given during CPR (aOR (95% CI) 2.2 (1.3–3.6), 1.9 (1.0–3.7), 14.6 (4.9–43.4), and 3.0 (1.4–6.4) respectively), whereas, age ≤ 52 years, bystander resuscitation, and initial rhythm documented (pulseless electrical activity and ventricular fibrillation) were independent predictors of survival to discharge (aOR (95% CI) 2.5 (0.9–6.5), 1.4 (0.5–3.8), 5.3 (1.5–18.4), and 3.1 (1.0–10.2) respectively).

**Conclusion:**

Our study notes that while the majority of arrests occur out of the hospital, only a small proportion of those arrests receive on-site CPR, which is a key contributor to unfavorable outcomes in this group. It is recommended that effective pre-hospital emergency care systems be established in developing countries which could potentially improve post-arrest outcomes. Younger patients, CPR initiation soon after arrest, presenting rhythm of pulseless ventricular tachycardia and ventricular fibrillation, and those requiring up to four shocks to revive are more likely to achieve favorable outcomes.

## Background

Cardiac arrest is a medical emergency characterized by abrupt cessation of cardiac mechanical function resulting in insufficient circulation of blood flow, as indicated by the absence of palpable central pulse and apnea, loss of pulse, blood pressure, and spontaneous respiration. Although the condition may be reversible with immediate intervention, it can lead to death if appropriate action is not taken promptly [1]. Basic life support consisting of emergency response system activation, cardiopulmonary resuscitation (CPR), and defibrillation with an automated external defibrillator as indicated by the American Heart Association’s guidelines are integral to the management of a cardiac arrest [2].

CPR is the attempt to restore circulation and maintain the viability of vital organs until the underlying cause for arrest can be addressed and definitive intervention can be initiated [3]. To achieve this goal, resuscitation is performed in multiple steps including chest compression, maintenance of airways, and rescue breaths or ventilation [2]. If performed successfully, *return of spontaneous circulation* (ROSC) is achieved. ROSC is defined as return of pulse and its maintenance for longer than 20 min. Another key outcome of CPR is *survival to discharge* (STD), which is variably defined as a patient transferred from ICU to ward, transferred from one facility to another, or discharged home from hospital under stable conditions. However, favorable outcomes are not always attained post-CPR. It is estimated that normal blood flow is restored in less than 30% of patients undergoing CPR [4]. Moreover, studies have demonstrated CPR to be a time- and resource-intensive practice, often described as a “violent, painful, and undignified” process for the patient [5].

The emergency department (ED) within a hospital primarily manages the highest volume of patients with cardiac arrest, either out-of-hospital arrests being brought to the ED or critically unwell patients brought to the ED who go on to arrest while receiving initial care in the ED. It is evident from existing literature that there are a number of factors that influence the outcome of CPR performed in the ED. These include patient characteristics such as age and gender, along with certain clinical and CPR-specific characteristics like cardiac arrest mechanism, initial rhythm documented after cardiac arrest, clinical setting, response time, and duration of CPR among others [6–8]. Hence, it is increasingly important to study pre-arrest and arrest parameters and have a better understanding of their prognostic implications so that high quality CPR can be applied in a rational, productive, and effective manner, on those patients that are most likely to benefit. Like most developing countries, Pakistan has poor patient medical record systems making it difficult to track and document outcomes of cardiac arrest within a hospital setting. Accurate assessment of CPR outcomes would supplant the paucity of information and allow us to make locally relevant predictions following cardiac arrest in resource-constrained settings like ours. This study aims to determine the outcome of CPR in cardiac arrest patients at a tertiary care hospital in Karachi, Pakistan, and identify predictors associated with positive outcomes.

## Methods

This retrospective study analyzed the medical records of all cardiac arrest patients who underwent CPR in the ED of The Indus Hospital (TIH), during an 8-year period from January 2008 to December 2015. TIH is tertiary care facility situated in Karachi, Pakistan, serving a large, middle-low income urban population. During the study period, this was a 150-bed facility with an ED comprising of 10 monitored beds and 2 resuscitation beds. Approximately 50,000 patients present to the ED per month, of which 10 to 15 patients either present with cardiac arrest or go into arrest during the course of ED stay.

Medical records of 800 patients that underwent CPR in the ED during the study period were included in this retrospective analysis. The inclusion criteria were patients aged 18 years and above who had sustained either an in- or out-of-hospital cardiac arrest and were brought to the ED for treatment. Patients with missing records or incomplete data, pronounced dead on arrival to the ED, those that left hospital against medical advice during the treatment process, those that were transferred out to another facility immediately for post-arrest care, and those with existing do-not-resuscitate orders were excluded from the final analysis. Similarly, patients that were shifted from the ED to the ICU/CCU but subsequently transferred within 24 h to another facility were excluded while analyzing predictors for STD due to unknown final outcomes. In addition, first event of cardiac arrest was taken as the seminal event in patients who had more than one episode of cardiac arrest. Thus, a total of 468 patients meeting the inclusion criteria were included in the study,

Patient demographics and clinical history were recorded to assess predictors associated with post-arrest outcomes. Clinical characteristics of cardiac arrest including location, possible cause of arrest, witnessed arrest, and initial rhythm were recorded. CPR characteristics including bystander-performed CPR, time interval from collapse to start of CPR, CPR duration, and number of shocks given were noted. For the purpose of this study, the CPR outcomes were determined to be ROSC and STD. STD category included those patients who were discharged home from the hospital as well as those patients that were transferred to another facility after a minimum stay of 24 h following ROSC.

### Data analysis

The data was analyzed using SPSS version 21.0 (IBM, Chicago, IL). For descriptive analysis, means and standard deviations were reported for continuous variables. As mentioned above, ROSC and STD were the outcomes of interest. Any association with a *P* value of 0.25 or less was included to build a parsimonious model using multiple logistic regression with backward stepwise elimination to test the significance at each step and assess the predictors for ROSC and STD. In addition, biologically or socially significant variables were included in the multiple logistic regression analysis. An odds ratio of more than 1 indicates an increased likelihood of the outcome (ROSC or STD), and *p* value < 0.05 was considered significant. Additionally, Yauden’s Index was used to determine cutoffs for patient’s age, collapse-to-start CPR time, and CPR duration for sustaining ROSC and STD separately.

## Results

**Fig. 1:**
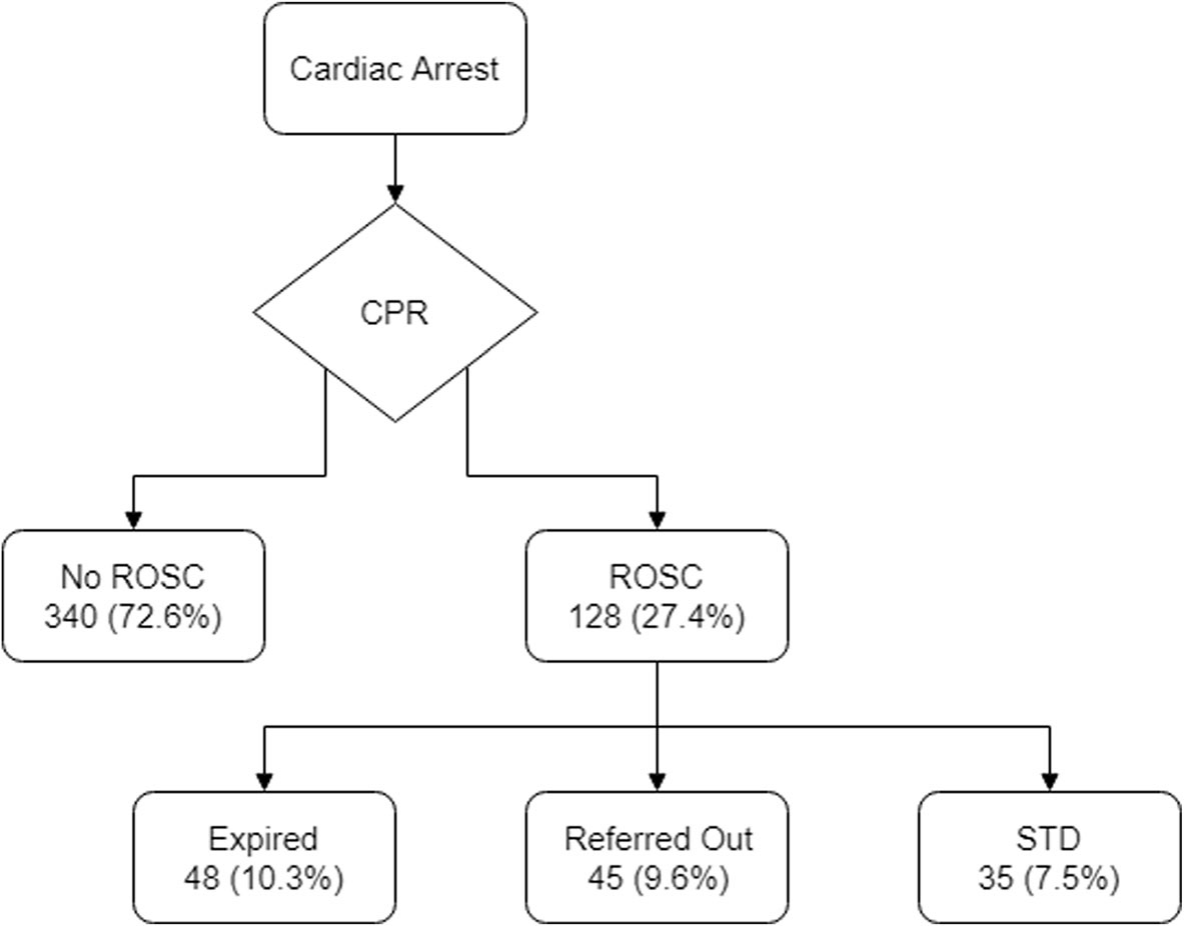
Outcome of cardiopulmonary resuscitation in cardiac arrest patients. This figure shows a breakdown of the study population, i.e., cardiac arrest patients that were presented to the emergency department of The Indus Hospital during the duration of the study period, based on the CPR outcomes

A total of 468 cardiac arrest patients who had CPR performed in TIH’s ED during the study period were included in this retrospective analysis. Out of the 468 patients included, ROSC was achieved by slightly more than a quarter (*n* = 128, 27.4%) of patients and STD was achieved in 35 (7.5%) patients (Table 1). Figure 1 provides details of the study population with regard to CPR outcomes. Demographics, clinical, and CPR characteristics of the patient population with stratification by ROSC and STD are detailed in Table 1.

**Table 1:** Characteristics of patients that sustained return of spontaneous circulation and those who survived till discharge

	Total (*n* = 468)	ROSC achieved (*n* = 128)	Survival to discharge (*n* = 35)
Age (years)			
Mean ± SD	56 ± 17	52 ± 18	49 ± 16
Median (range)	60 (18–97)	53 (18–97)	48 (18–80)
Gender, *n* (%)			
Male	287 (61)	71 (55)	21 (60)
Female	181 (39)	57 (45)	14 (40)
No. of co-morbidities, *n* (%)			
No	142 (30)	34 (27)	7 (20)
One	144 (31)	43 (33)	13 (37)
Two	142 (30)	44 (34)	13 (37)
Greater than 2	40 (9)	7 (6)	2 (6)
CPC score, n(%)			
≤ 2	452 (97)	124 (98)	34 (97)
> 2	15 (3)	3 (2)	1 (3)
Initial cause of arrest, *n* (%)			
Cardiopulmonary	302 (65)	74 (58)	21 (60)
Non-cardiopulmonary	166 (35)	54 (42)	14 (40)
Location of arrest, *n* (%)			
In-hospital (ED)	154 (33)	62 (48)	22 (63)
Out-of-hospital	314 (67)	66 (52)	13 (37)
Witnessed arrest, *n* (%)	279 (60)	110 (86)	31 (89)
Bystander performed CPR, *n* (%)	158 (34)	67 (52)	22 (63)
First documented rhythm, *n*			
Non-shockable, *n* (%)	351	68	17
Asystole	185 (40)	25 (20)	3 (9)
Pulseless electrical activity	166 (35)	43 (34)	14 (40)
Shockable, *n* (%)	117	60	18
Ventricular fibrillation	81 (17)	43 (34)	15 (42)
Pulseless ventricular tachycardia	36 (8)	17 (13)	3 (9)
No. of shocks given, *n* (%)			
0	351 (75)	67 (52)	17 (49)
1–4	58 (12)	38 (30)	11 (31)
> 4	59 (13)	23 (18)	7 (20)
Interval between collapse to start CPR (min), *n* (%)			
Mean ± SD	30 ± 22	15 ± 12	13 ± 12
Median (range)	30 (1–90)	10 (1–60)	10 (1–60)
CPR duration (min), *n* (%)			
Mean ± SD	20 ± 9	22 ± 9	21 ± 9
Median (range)	20 (5–50)	20 (5–40)	20 (10–40)
Doctors shift, *n* (%)			
Morning	179 (38)	54 (42)	14 (40)
Evening	105 (22)	27 (21)	9 (26)
Night	184 (39)	47 (37)	12 (34)
Team leader, *n* (%)			
Resident	311 (66)	85 (66)	24 (69)
Staff	157 (34)	43 (34)	11 (31)
Initial serum glucose, *n* (%)			
Mean ± SD	185 ± 125	170 ± 106	151 ± 91
Median (range)	133 (30–660)	133 (30–477)	127 (30–456)
Epinephrine, *n* (%)			
Less than 5 ampoules	315 (67)	73 (57)	20 (57)
5 or more ampoules	153 (33)	55 (43)	15 (43)

Of the patients achieving ROSC and STD, a higher proportion were males (55% and 60% respectively). Younger patients had a significantly better chance of sustaining ROSC (*p* = 0.004) and achieving STD in comparison with older patients (*p* = 0.386). While the mean age of our patient group was 56 years, the mean age of patients that sustained ROSC was 52 years and that of STD was even lower, at 49 years.

As demonstrated by the results, in-hospital arrests were more likely to achieve ROSC and eventually STD when compared to out-of-hospital arrests. In our population, 314 (67%) patients suffered a cardiac arrest prior to their arrival to the hospital’s ED, while the remaining 154 (33%) patients had an arrest in the ED during treatment for another presenting complaint. Of the 314 patients that had an out-of-hospital arrest, 66 (21%) achieved ROSC and 13 (4%) STD. In comparison, of the 154 patients that had an in-hospital arrest, 62 (40%) patients achieved ROSC and 22 (14%) patients STD. Of the 314 patients that had an out-of-hospital arrest, nearly half (*n* = 138, 44%) of the events were witnessed. In only 23 of these events, CPR was performed by a bystander (17% of witnessed arrests, and 7% of overall OHCA subset).

In terms of the types of initial cardiac arrest rhythms, the 117 patients with shockable rhythms (pulseless ventricular tachycardia (VT) and ventricular fibrillation (VF)) had a significantly higher chance of achieving ROSC (51.3%) and STD (42.9%), compared to non-shockable rhythms (19.4% and 41.5%, *p* < 0.0001, 0.898 respectively). The number of shocks received by patients with shockable rhythms ranged from two to nine, with no patient achieving ROSC after the eighth shock. Outcomes were more favorable in patients receiving four or less shocks during resuscitation (65.5% ROSC and 42.3% STD) as compared to more than four shocks (39% ROSC and 43.8% STD, *p* < 0.0001, 0.988 respectively). In the latter category, ROSC was achieved after five shocks (*n* = 6), six shocks (*n* = 11), and eight shocks (*n* = 6). Similarly, the seven patients that achieved STD in this category did so after five shocks (*n* = 2), six shocks (*n* = 2), and eight shocks (*n* = 3).

When assessing for the predictors of ROSC, in the final multiple logistic regression model, patients’ age (≤ 49 years aOR 2.2; 95% CI 1.3–3.6, reference > 49 years), as well as if it was a witnessed arrest (aOR 1.9; 95% CI 1.0–3.7, reference unwitnessed arrest), along with the time interval between collapse-to-start CPR (≤ 30 min OR 14.7; 95% CI 4.9–43.4, reference > 30 min) and number of shocks given during CPR (1–4 aOR 3.0; 95% CI 1.4–6.4, reference > 4 shocks) were positively associated with ROSC (see Table 2).

**Table 2:** Assessing for the predictors associated with return of spontaneous circulation (*N* = 128)

	ROSC achieved^a^	
	Unadjusted OR (CI)^b^	Adjusted OR (CI)^c^
Age groups		
≤ 49	2.2 (1.5–3.4)**	2.2 (1.3–3.6)*
> 49	Ref	Ref
Patient’s gender		
Females	1.4 (0.9–2.1)	–
Males	Ref	Ref
Co-morbidities		
No co-morbidities	Ref	Ref
One co-morbidity	1.4 (0.8–2.3)	–
Two co-morbidities	1.4 (0.8–2.4)	–
More than two co-morbidities	0.7 (0.3–1.7)	–
Location of cardiac arrest		
In-hospital (ED)	2.5 (1.7–3.9)**	–
Out-of-hospital	Ref	Ref
Witnessed arrest		
Yes	6.2 (3.6–10.6)**	1.9 (1.1–3.7)*
No	Ref	Ref
Cause of cardiac arrest		
Cardiopulmonary	Ref	Ref
Non-Cardiopulmonary	1.5 (0.1–2.3)	–
Initial cardiac rhythm during arrest		
Shockable	4.4 (2.8–6.9)**	–
Non-shockable	Ref	Ref
Rhythm documented at the time of initiation of CPR:		
Asystole	Ref	Ref
Pulseless electrical activity	2.2 (1.3–3.9)*	–
Ventricular fibrillation	7.2 (4.0–13.3)**	–
Pulseless ventricular tachycardia	5.7 (2.6–12.5)**	–
CPC score before cardiac arrest		
≤ 2	1.5 (0.4–5.4)	–
> 2	Ref	Ref
Bystander performed CPR		
Yes	3.0 (2.0–4.6)**	–
No	Ref	Ref
Time interval from collapse-to-start CPR duration		
≤ 30 min	28.2 (10.2–78.1)**	14.6 (4.9–43.4)**
> 30 min	Ref	Ref
CPR duration		
< 20 min	Ref	Ref
≥ 20 min	1.656 (1.081–2536)*	–
Number of shocks given		
0	0.4 (0.2–0.7)*	0.8 (0.4–1.4)
1–4	3.0 (1.4–6.3)*	3.0 (1.4–6.4)*
> 4	Ref	Ref
Epinephrine		
< 5 ampules	Ref	Ref
≥ 5 ampules	1.9 (1.2–2.8)*	–
Initial serum glucose		–
Doctor’s shift		
Morning	1.3 (0.8–2.0)	–
Evening	1.0 (0.6–1.7)	–
Night	Ref	Ref
Team leader		
Staff	1.0 (0.7–1.5)	–
Resident	Ref	Ref

^a^ROSC not achieved is the reference category, ^b^Univariate binary logistic regression, ^c^Multivariate binary logistic regression.*p value< 0.05, **p value< 0.0001

Similarly, to assess predictors associated with STD, in the final multiple logistic regression model, patients’ age (≤ 52 years aOR 2.5; 95% CI 0.9–6.5, reference > 52 years), as well as bystander resuscitation (aOR 1.4; 95% CI 0.5–3.8, reference no bystander performed CPR), and initial rhythm documented (PEA aOR 5.3; 95% CI 1.5–18.4, VF aOR 3.1; 95% CI 1.0–10.2, reference asystole) were statistically significant predictors for STD (see Table 3).

**Table 3:** Assessing for the predictors associated with survival to discharge (*N* = 35)

	Survived to discharge^a^	
	Unadjusted OR (CI)^b^	Adjusted OR (CI)^c^
Age groups		
≤ 52	2.0 (0.8–4.9)	2.5 (0.9–6.5)
> 52	Ref	Ref
Patient’s gender		
Females	0.9 (0.4–2.1)	–
Males	Ref	Ref
Co-morbidities		
No co-morbidities	Ref	Ref
One co-morbidity	1.3 (0.4–4.3)	–
Two co-morbidities	1.3 (0.4–4.3)	–
More than two co-morbidities	1.7 (0.2–15.0)	–
Location of cardiac arrest		
In-hospital (ED)	1.6 (0.6–3.8)	–
Out-of-hospital	Ref	Ref
Witnessed arrest		
Yes	1.1 (0.3–4.3)	–
No	Ref	Ref
Cause of cardiac arrest		
Cardiopulmonary	Ref	Ref
Non-cardiopulmonary	1.0 (0.4–2.3)	–
Initial cardiac rhythm during arrest		
Shockable	1.1 (0.4–2.5)	–
Non-shockable	Ref	Ref
Rhythm documented at the time of initiation of CPR:		
Asytole	Ref	Ref
Pulseless electrical activity	5.5 (1.3–24.3)*	5.3 (1.5–18.4)*
Ventricular fibrillation	4.1 (1.0–17.1)*	3.1 (1.0–10.2)
Pulseless ventricular tachycardia	1.6 (0.3–10.1)	–
CPC score before cardiac arrest		
≤ 2	1.5 (0.1–17.0)	–
> 2	Ref	Ref
Bystander performed CPR		
Yes	1.3 (0.5–3.2)	1.4 (0.5–3.8)
No	Ref	Ref
Time interval from collapse-to-start CPR duration		
≤ 10 min	1.8 (0.7–4.5)	–
> 10 min	Ref	Ref
CPR duration		
< 30 min	Ref	Ref
≥ 30 min	1.1 (0.5–2.9)	–
Number of shocks given		
0	0.9 (0.3–2.9)	–
1–4	0.9 (0.3–3.3)	–
> 4	Ref	Ref
Epinephrine		
< 5 ampules	Ref	Ref
≥ 5 ampules	1.3 (0.5–1.0)	–
Initial serum glucose	1.0 (0.9–1.1)	–
Doctor’s shift		
Morning	1.3 (0.5–3.5)	–
Evening	2.0 (0.6–6.5)	–
Night	Ref	Ref
Team leader		
Staff	0.8 (0.3–2.1)	–
Resident	Ref	Ref

^a^Died is the reference category, ^b^Univariate binary logistic regression, ^c^Multivariate binary logistic regression.*p value < 0.05, **p value< 0.0001

## Discussion

Cardiac arrest is a common medical emergency that requires prompt intervention that usually includes CPR. A meta-analysis of 51 studies of in-hospital cardiac arrests from western countries was published in 1998, where STD rates ranging between 13.4 and 14.6% were reported; similar results have been noted more recently [9, 10]. A study from Malaysia that evaluated the outcome of CPR reported ROSC of 30.2% and STD of 9.5% in patients undergoing cardiac arrest [7]. It is difficult to determine CPR outcomes in developing countries since studying cardiac arrest and factors influencing outcomes requires a combination of pre-hospital, ED, and inpatient data, which is challenging to obtain in most LMIC settings. Furthermore, in order to achieve optimum outcomes following CPR, it is essential for high-quality CPR to be administered in a pre-hospital setting on out-of-hospital cardiac arrest (OHCA) patients. However, it is difficult to ascertain the quality of bystander CPR and hence study its effect on survival; this remains a key challenge. Retrospective studies of CPR outcomes in tertiary care facilities across Pakistan found ROSC rates ranging from 35 to 72% and STD rates between 11 and 22% [11–14]; however, factors influencing these outcomes have not been reported in any of these studies. Comparable results have been reported from India [15, 16] where the context is very similar to that seen in Pakistan. Our study showed ROSC and STD rates of 27% and 7.5% respectively, which is lower than those reported in other studies from the region. However, when we look at the ROSC and STD rates for only the in-hospital arrest subset in our study, the rates are 40% and 14% respectively, which compare well with reported data.

It is well documented that immediate initiation of CPR improves outcomes significantly [2, 17–22]. Countries reporting successful resuscitation of cardiac arrest patients have effective systems in place to assist and transport patients, effective hotline centers, well-equipped ambulances, and highly skilled and experienced pre-hospital care teams [23–28], which inevitably translate into better overall outcomes. In contrast, in Pakistan and most other LMICs, basic life support training for lay persons is virtually non-existent and pre-hospital ambulance and paramedical care is limited even in urban settings, resulting in delayed response times. Most patients are transported to hospitals in private vehicles by non-medical personnel and do not receive even the most basic of life support care in the critical pre-hospital phase [29–31]. Moreover, the effectiveness of any first-line care that may be administered is questionable. It is therefore not surprising that our data shows significantly poorer outcomes among patients suffering cardiac arrest outside the hospital; only 7% of 314 patients suffering arrest outside of our ED received pre-hospital CPR, and the ROSC and STD rates were 21% and 4% respectively. Mean duration of 38 min (SD ± 20) to initiate CPR among the OHCA subset that exceeded the 30 minutes cut-off for ROSC and 10 minutes cut-off for STD provided by the Yauden’s Index. The enormity of the challenge is amplified when we see that most patients suffer cardiac arrests before reaching a hospital; 67% of our study population had suffered an arrest prior to presentation to our ED.

Studies have demonstrated how advancing age is associated with decreased likelihood of survival to hospital discharge, whereas others argue that is does not exert any significant effect on the outcome of CPR [12, 13]. The mean age in the overall, ROSC, and STD groups was 56, 52, and 49 years respectively, indicating that younger age is associated with better outcomes post-CPR. Age as an independent prognostic factor on expected outcome may be helpful in counseling families during and following CPR.

Cardiac arrest in adults is associated with an initial rhythm of VF or VT in most cases and degenerates to asystole with time [14, 32, 33]. In comparison, among our study population, asystole was the most common cardiac rhythm documented at the start of CPR (*n* = 185; 40%), followed by pulseless electrical activity (PEA), VF, and VT; this may well reflect a delayed initiation of CPR in our setting for reasons detailed above. Studies reviewed suggest immediate survival (i.e., ROSC) is better in patients presenting with shockable rhythms [7, 8, 34–36]; this is supported by our findings with significantly better ROSC and STD rates seen in patients that had a shockable initial rhythm (51% and 15%) as compared to those presenting with non-shockable rhythms (19% and 4% respectively). However, multivariate regression analysis found that patients with an initial documented rhythm of PEA and VF were three to five times more likely to survive to discharge as compared to asystolic cardiac rhythm. The majority of patients included in this analysis experienced an out-of-hospital arrest where the initial rhythm was not recorded; the rhythm from the time when the patient is first seen in the ED is taken as the first recorded rhythm in this study, which may make a true interpretation difficult. Additionally, studies have shown prompt defibrillation to be associated with improved survival rates and good neurological outcomes post-CPR. Hence, the availability of automated external defibrillators in public spaces is recommended to analyze heart rhythms and advises rescuers/bystanders to administer shocks in a timely fashion, without significant delays among patients having a shockable, initial cardiac rhythm.

The optimum number of shocks administered in our study ranges between two and four, which is almost three times more likely to be associated with initial survival. An increased requirement beyond this cutoff of four shocks during the course of CPR indicates that the patient is not responding to defibrillation, either because of a delayed presentation or because of the underlying pathology. Evidence-based guidelines would help the CPR teams to realize that efforts beyond a certain number of shocks would be unlikely to yield favorable outcome and therefore help in deciding when to cease CPR. From our retrospective data, it was difficult to determine the quality of chest compressions provided and the time interval between shocks. A prospective study could better elucidate the impact of these parameters on outcomes.

There is little consensus in existing literature on duration of CPR that is associated with optimal outcomes. Good outcomes have been reported with CPR duration of up to 10 min in patients undergoing an in-hospital cardiac arrest [37, 38]. Ishtiaq et al. showed a mean CPR duration of 15 min (SD ± 10) in those patients surviving to discharge. In a large multicenter study of data from 435 hospitals, Goldberger et al. demonstrated that a median CPR duration of 25 min (IQR 25–28 min) correlated with the best rates of ROSC. In our study, we found that the overall mean duration for CPR was 20 min (SD ± 9); this did not differ significantly for the ROSC and STD groups. This also did not vary when stratified for arrests occurring prior to or during ED stay. Based on our findings and those of other authors described above, we recommend that CPR be continued for up to 30 min for all patients presenting with cardiac arrest.

### Limitations

For the majority of patients who sustained an arrest out of hospital, accurate information about the event is very difficult to record. For the in-hospital parameters, we had to rely on data entered in medical records over an 8-year period by a large number of different providers. This inevitably led to variability in the way the information was recorded. Patient data beyond the hospital stay was not available for many patients, and hence, correlation of CPR with longer term benefit and survival could not be studied. Future prospective studies should be conducted allowing standardized documentation of events and prognostic factors allowing a more accurate understanding of the determinants of CPR outcomes.

## Conclusions

The present study highlighted that improved CPR outcomes are associated with younger age, CPR duration not exceeding 30 min, and if the rhythm is determined to be shockable, the optimum number of shocks given to range between one and four. If the pre-arrest status of a patient can be determined based on locally relevant parameters, the formulation of rapid response teams can be quickly implemented to stabilize the patient in a timely manner.

### Recommendations

Low literacy rates in the population combined with a general a lack of awareness and training on emergency response limit the ability to provide on-site basic life support in Pakistan. A poor or non-existent ambulance service across the country leads to inadequate emergency care provision at the site of event or during transfer which further compromises pre-hospital care. The lack of an organized and ongoing training curriculum for facility-based EM personnel is also a contributing factor. And finally, the lack of coordination between the components of emergency care leads to sub-optimal care pathways and hence poor outcomes. We recommend a multi-layered approach addressing gaps at all levels. Community mobilization through grass-root organizations and religious and youth leaders would encourage local ownership and engagement. High school children have demonstrated good retention of basic life support skills in a pilot study in Pakistan [39]. Medical students have proven to be highly motivated and resourceful network, with several groups providing first responder training to laypersons in different parts of the country. These efforts should be carried out in conjunction with an awareness campaign for the general public on the importance of learning and administering of basic life support in addition to summoning trained EM personnel in a timely manner. A centrally controlled ambulance service with trained personnel could help provide high-quality on-site and during transfer care, allowing the patient to reach the facility in an optimized state. It is recommended that a sound theoretical knowledge along with practical training in CPR should be an integral part of the curriculum for all medical personnel, particularly those at the front line including emergency departments, with regular refresher trainings. Pakistan has a strong philanthropic base which is already engaged in bridging gaps in public sector health services across the country; coordination among stakeholders would feasibly lead to better coordinated emergency care pathway with improved outcomes.

## Abbreviations

CPR: Cardiopulmonary resuscitation
ED: Emergency department
IHCA: In-hospital cardiac arrest
OHCA: Out-of-hospital cardiac arrest
PEA: Pulseless electrical activity
ROSC: Return of spontaneous circulation
STD: Survival to discharge
TIH: The Indus Hospital
VF: Ventricular fibrillation
VT: Pulseless ventricular tachycardia
